# Angiogenic role of miR-20a in breast cancer

**DOI:** 10.1371/journal.pone.0194638

**Published:** 2018-04-04

**Authors:** Gines Luengo-Gil, Enrique Gonzalez-Billalabeitia, Sergio Alejo Perez-Henarejos, Esther Navarro Manzano, Asuncion Chaves-Benito, Elena Garcia-Martinez, Elisa Garcia-Garre, Vicente Vicente, Francisco Ayala de la Peña

**Affiliations:** 1 Department of Hematology and Medical Oncology, Hospital Universitario Morales Meseguer y Centro Regional de Hemodonación, Murcia, Spain; 2 Department of Internal Medicine, University of Murcia, Murcia, Spain; 3 IMIB-Arrixaca, Murcia, Spain; 4 Universidad Católica San Antonio de Murcia (UCAM), Murcia, Spain; 5 Department of Pathology, Hospital Universitario Morales Meseguer, Murcia, Spain; University of South Alabama Mitchell Cancer Institute, UNITED STATES

## Abstract

**Background:**

Angiogenesis is a key process for tumor progression and a target for treatment. However, the regulation of breast cancer angiogenesis and its relevance for clinical resistance to antiangiogenic drugs is still incompletely understood. Recent developments on the contribution of microRNA to tumor angiogenesis and on the oncogenic effects of miR-17-92, a miRNA cluster, point to their potential role on breast cancer angiogenesis. The aim of this work was to establish the contribution of miR-20a, a member of miR-17-92 cluster, to tumor angiogenesis in patients with invasive breast carcinoma.

**Methods:**

Tube-formation in vitro assays with conditioned medium from MCF7 and MDA-MB-231 breast cancer cell lines were performed after transfection with miR-20a and anti-miR20a. For clinical validation of the experimental findings, we performed a retrospective analysis of a series of consecutive breast cancer patients (n = 108) treated with neoadjuvant chemotherapy and with a full characterization of their vessel pattern and expression of angiogenic markers in pre-treatment biopsies. Expression of members of the cluster miR-17-92 and of angiogenic markers was determined by RT-qPCR after RNA purification from FFPE samples.

**Results:**

*In vitro* angiogenesis assays with endothelial cells and conditioned media from breast cancer cell lines showed that transfection with anti-miR20a in MDA-MB-231 significantly decreased mean mesh size and total mesh area, while transfection with miR-20a in MCF7 cells increased mean mesh size. MiR-20a angiogenic effects were abrogated by treatment with aflibercept, a VEGF trap. These results were supported by clinical data showing that mir-20a expression was higher in tumors with no estrogen receptor or with more extensive nodal involvement (cN2-3). A higher miR-20a expression was associated with higher mean vessel size (*p* = 0.015) and with an angiogenic pattern consisting in larger vessels, higher VEGFA expression and presence of glomeruloid microvascular proliferations (*p*<0.001). This association was independent of tumor subtype and VEGFA expression.

**Conclusions:**

Transfection of breast cancer cells with miR-20a induces vascular changes in endothelial tube-formation assays. Expression of miR-20a in breast invasive carcinomas is associated with a distinctive angiogenic pattern consisting in large vessels, anomalous glomeruloid microvascular proliferations and high VEGFA expression. Our results suggest a role for miR-20a in the regulation of breast cancer angiogenesis, and raise the possibility of its use as an angiogenic biomarker.

## Introduction

Breast cancer is the main cause of cancer-related mortality among women from developed countries. Although its prognosis has improved in recent years, with five-year survival over 80%, development of distant metastasis occurs in around 30% of cases after treatment with surgery and adjuvant systemic therapy [1]. New and improved treatments are now available for both early and metastatic breast cancer, but more active targeted therapies are still needed for women with breast cancer. Tumor angiogenesis, one of the hallmarks of cancer, is a key process for tumor progression and metastases. A vast amount of clinical and experimental data has demonstrated the prognostic relevance and the biological implications of angiogenic activation in breast cancer. In particular, VEGFA (vascular and endothelial growth factor A) has a key role in breast cancer progression, through its effects on tumor angiogenesis, and also through its autocrine functions in migration, invasion and survival of breast cancer cells [2]. An association of VEGFA expression with early recurrence and worse survival of breast cancer has been previously demonstrated, although current data do not support the use of VEGFA levels as a biomarker for guiding antiangiogenic therapy [3,4]. Partly due to the lack of validated biomarkers, the true impact of therapeutic strategies targeting angiogenesis for breast cancer has been below the initial expectations [5,6]. The introduction of antiangiogenic drugs in the adjuvant or neoadjuvant setting, corresponding to earlier stages of breast cancer, has also rendered poor results, although more studies are needed [7,8]. Additionally, modulation of tumor angiogenesis by conventional chemotherapy, with potential synergistic and antagonistic effects, further confounds the interpretation of clinical results. In fact, others and we have demonstrated that chemotherapy exerts variable changes in tumor vascular network, which are clearly related neither to pathologic response nor to patients outcome [9–11].

A major problem for understanding the limited antiangiogenic effects of antiangiogenic therapy for breast cancer is the lack of validated predictive biomarkers. Besides the highly variable results with circulating biomarkers, the impact of angiogenesis-targeted drugs and chemotherapy on the usual markers of angiogenesis, such as microvessel density (MVD) or vascular endothelial growth factor A (VEGFA) expression has been inconsistent in the clinical setting [12,13]. Some translational studies suggest that vascular characteristics, related to vessel size and morphology, are associated with prognostic differences in breast cancer. Specifically, a higher vascular size and complexity, experimentally linked to thrombospondin and VEGFA changes, seem to confer a worse prognosis in breast cancer, as others and we have shown before [9,14]. In particular, we have previously characterized a bad-prognosis VEGFA-related angiogenic profile characterized by a higher mean vascular size (MVS) and by the presence of glomeruloid microvascular proliferation (GMP), but without increased microvascular density (MVD). This vascular profile is associated to high expression of VEGFA [9].

The regulation of angiogenesis in breast cancer depends, as in other malignant neoplasms, on a complex network of interactions between stromal and tumor cells, which modifies endothelial cell function and the levels of pro- and anti-angiogenic factors in the tumor microenvironment. In the current active search for unveiling angiogenesis mechanisms, recent data support the potential role of microRNA in the regulation of tumor angiogenic balance [15,16]. MicroRNA are short RNA chains that regulate gene expression either by inhibition or degradation of mRNA or by direct interference with protein translation. Several miRNA are able to target pro-angiogenic or anti-angiogenic mediators both in the tumor and endothelial cells, as well as in other cells of the peritumoral stroma. In particular, the cluster miR-17-92, also known as OncomiR-1 for its oncogenic effects, is expressed in breast cancer, with a higher expression in hormone receptor (ER) negative tumors. The highly conserved polycistronic cluster miR-17-92 includes six different mature miRNAs (miR-17, miR-18a, miR-19a, miR-19b, miR-20a and miR-92a), and there are also two paralog clusters (miR-106b-363 and miR-106b-25). According to seed sequences, three families have been defined: miR-17 (miR-17, miR-20a, miR-18a), miR-19 (miR-19a and miR-19b) and miR-92. The expression of miR-17-92 depends, at least partially, on oncogenes MYC and KRAS, and is also related to estrogen receptor (ER) pathway in breast carcinomas. Some members of the cluster induce pro-angiogenic effects, while other miRNAs may show different actions, probably depending of the cell context, but experimental data in lymphoma [17] and other tumors support a predominantly pro-angiogenic effect [18,19]. Several validated targets (THBS1 or thrombospondin 1, VEGFA, TIMP) are relevant for the vascular effects of the cluster. Indirect effects through modulation of coagulation factors, such as tissue factor, or through inhibition of TFG-beta signaling [19,20], might also impact on tumor angiogenesis. Conversely, VEGFA may upregulate miR-17-92 expression in endothelial cells thereby mediating postnatal angiogenesis [21]. A recent work on miR-17-92 has also demonstrated its role in endothelial proliferation and sprouting in the context of VEGF-dependent tumor angiogenesis. These experimental data point to the potential value of miR-17-92 as a target for blocking VEGFA-stimulated tumor angiogenesis [22]. However, no clinical or experimental studies are available addressing the relevance of miR-17-92 for breast cancer angiogenesis, which is highly dependent on VEGF stimulation.

The aim of our work was to determine the contribution of miR-20, a member of miR-17-92 cluster, to breast cancer angiogenesis, first analyzing the angiogenic effects of its expression on breast cancer cell lines, and second determining its association with the angiogenic characteristics of a clinical series of breast carcinomas.

## Patients and methods

### Patients and treatment

We analyzed a group of consecutive breast cancer patients (n = 108) previously included in a study of angiogenesis in the neoadjuvant chemotherapy setting, for whom a full characterization of vessel profile (microvascular density [MVD], mean vessel size [MVS], glomeruloid microvascular proliferation [GMP]) and angiogenic markers expression (VEGFA, PDGFA, THBS1, HIF1a) was available (n = 86) [9]. All patients had a histological diagnosis of breast cancer and had been treated with neoadjuvant chemotherapy (most of them sequential anthracyclines and taxanes) at the Hospital General Universitario Morales Meseguer, Spain. The study was conducted according to the principles expressed in the Declaration of Helsinki. All patients gave written informed consent. This specific study was approved by the Institutional Review Board of Hospital Morales Meseguer (Comité Etico y de Investigación Clínica del Hospital G. Universitario Morales Meseguer), with internal code of approval: ESTU-23/12.

### Histological studies

Assessment of vascular profile was performed in 4 μm full-section pre-treatment core-biopsies, previously stained with an anti-CD34 antibody (Clone QBEnd-10; M7165; DAKO, Glostrup, Denmark), as previously reported. After automatized digital scanning (Leica SCN400F), three to five 100x digital pictures were independently obtained by two of the authors. Computerized image analysis based on ImageJ software (NIH) was used to determine microvessel density (normalized to a 0.20 mm field) and mean vessel size (vessel area per vessel; μm^2^). Manual quantification of glomerular vascular proliferation (GMP) was also performed; the presence of at least one GMP was used as a criterion to define a case as GMP+.

### RNA purification and RT-qPCR

Total RNA from FFPE pre-treatment biopsies was extracted using RNeasy FFPE Kit (QIAGEN, Germantown, MD, USA) according to supplier instructions. Total RNA from cultured cells was purified using RNAzol reagent (MRC, Cincinnati, OH, USA) and Direct-zol RNA MiniPrep Kit (ZYMO Research, Irvine, CA, USA). RT-qPCR for all microRNA and for mRNA (with pre-amplification) was performed using TaqMan Gene expression Assays (Fisher Scientific, Madrid, Spain) in a LightCycler 480® Real-Time PCR System. Expression studies for transfected cell lines were performed 48 hours after transfection. Relative expression was calculated as 2^ΔCT^ using U6 snRNA and ACTB as endogenous controls for microRNA and mRNA respectively.

To determine VEGF effect on miR-20a expression by luminal breast cancer cells, MCF7 cells were grown in absence or presence of recombinant human VEGF (Fisher Scientific, Madrid, Spain) at low and high concentrations (0.5 ng/ml and 10 ng/ml) over 24 or 48 hours. After incubation, cells were washed twice with PBS and lysed with RNAzol (MRC, Cincinnati, OH, USA). RNA was extracted and miR-20a expression was evaluated as previously described.

### miRNA transfections

MCF7 (ATCC: HTB-22) and MDA-MB-231 (ATCC: CRM-HTB-26) were provided by Servicio de Apoyo a la Investigación (SAI, Universidad de Murcia), and maintained in DMEM-GlutaMAX 1 g/l glucose, 10% FBS (GIBCO, Madrid, Spain). Cell line authentication testing was determined in our lab by analysis of the ATCC recommended STR loci (TH01, TPOX, vWA, CSF1PO, D16S539, D7S820, D13S317 and D5S818 plus Amelogenin). MiR-20a transient transfections were performed using siPORT NeoFX Transfection Agent (Invitrogen, Barcelona, Spain) and *mirVana™ miRNA Mimics* (Life Technologies) or miRCURY LNA™ microRNA Inhibitors (EXIQON, Vedbaek, Denmark) according to manufacturer’s instructions in 6-well culture plates. Each experiment was repeated three times with at least three replicates for each condition.

### *In vitro* angiogenesis assays

For culture medium preparation, after 24 hours of transient transfection in 6-well plates (200,000 cells per well), cells were washed twice with PBS and then 3 ml of fresh medium (DMEM 1g/l glucose, 1% non-essential aminoacids, 1% pyruvate, without serum and without phenol red) was added. After 24 hours of cell growing, conditioned medium was collected, centrifuged at 200g for 5 minutes, and then concentrated 10x using Vivaspin 20 (10,000 MWCO) concentrators (Sartorius, Madrid, Spain), 0.22 μm filtered and stored at -80°C until use. For all *in vitro* angiogenesis assays, fetal bovine serum was absent of cell culture medium.

For angiogenesis assays, 5.000 EA.hy926 endothelial cells and 100 μl conditioned medium from either MCF7 or MDA-MB-231 transfected with miR-20a *mimic*/*silencer* and controls (negative control transfected cells) was added (8 replicates per condition) to 96-well plates containing BD Matrigel™ Basement Membrane Matrix not reduced in growth factors (Becton Dickinson, Madrid, Spain). After 24 hours of incubation with serum-free medium, the length and characteristics of vessel-like tubes, including the number of nodes or branchpoints, were measured by image analysis with Angiogenesis Analyzer plugin for ImageJ [23]. Each experiment was repeated three times.

For evaluating miR-20a effect on angiogenesis in a VEGF-rich or VEGF-free environment, conditioned medium from MCF7 was incubated with 500 μg/ml Aflibercept (Zaltrap, Barcelona, Spain) or PBS control for 1 hour at 37°C and then *in vitro* angiogenesis assays were performed as previously described.

Evaluation of endothelial cell proliferation after 24 hours growing in conditioned medium (without fetal bovine serum) from MCF7 cells was assayed by XTT Cell Proliferation Kit II (Roche, Madrid, Spain) in 96-well plates (five well per condition) in a Biotek Synergy HT reader. The conditions assayed were: conditioned medium from MCF7 previously transfected with miR-20a or scrambled control (50 nM each) ± aflibercept (500 μg/ml) (Sanofi-Aventis, Spain).

### Electrophoresis and western blotting

After cell lysis using RIPA buffer with phosphatase and protease inhibitors (Thermo-Fisher) protein concentration was determined by bicinchoninic acid assay. SDS-PAGE was carried out with 25 μg of whole protein lysates per well in a Bio-Rad electrophoresis system. Transference was performed using Amersham TE 77 PWR for one hour. Western-blot was performed using 5% milk in PBST as blocking agent and VEGFA (#ab46154, Abcam, Cambridge, UK) and β-actin (#A5441; Sigma-Aldrich, Madrid, Spain) primary antibodies. PVDF membranes were revealed with Amersham™ ECL™ Prime in ImageQuant LAS4000 (GE-Healthcare, Murcia, Spain).

### Statistical analyses and external validation of results

Statistical analysis was performed with SPSS 21.0 software (SPSS, Inc., Chicago, IL, USA). Non-parametric tests (U Mann-Whitney or Kruskal-Wallis) were used for comparison of quantitative variables between groups, and Chi-squared test for comparison of proportions. The association between continuous variables was tested with the Spearman rank-correlation coefficient. To evaluate the contribution of miR-20a to a high-risk angiogenic profile, we built multivariate logistic regression models including VEGFA expression and triple-negative subtype as covariates. Survival analysis with Kaplan-Meier method and log-rank test was performed to evaluate the impact of miRNA expression on overall (OS) and disease-free survival (DFS). All test were two-sided and a *P* value ≤ 0.05 was considered as statistically significant.

External validation of angiogenesis markers and miRNA expression correlation and of associations between miRNA expression and clinical characteristics was based on the analysis of breast cancer public database from TCGA (The Cancer Genome Atlas) [24].

## Results

### *In vitro* effects of miR-20a on breast cancer angiogenesis

**Fig 1:**
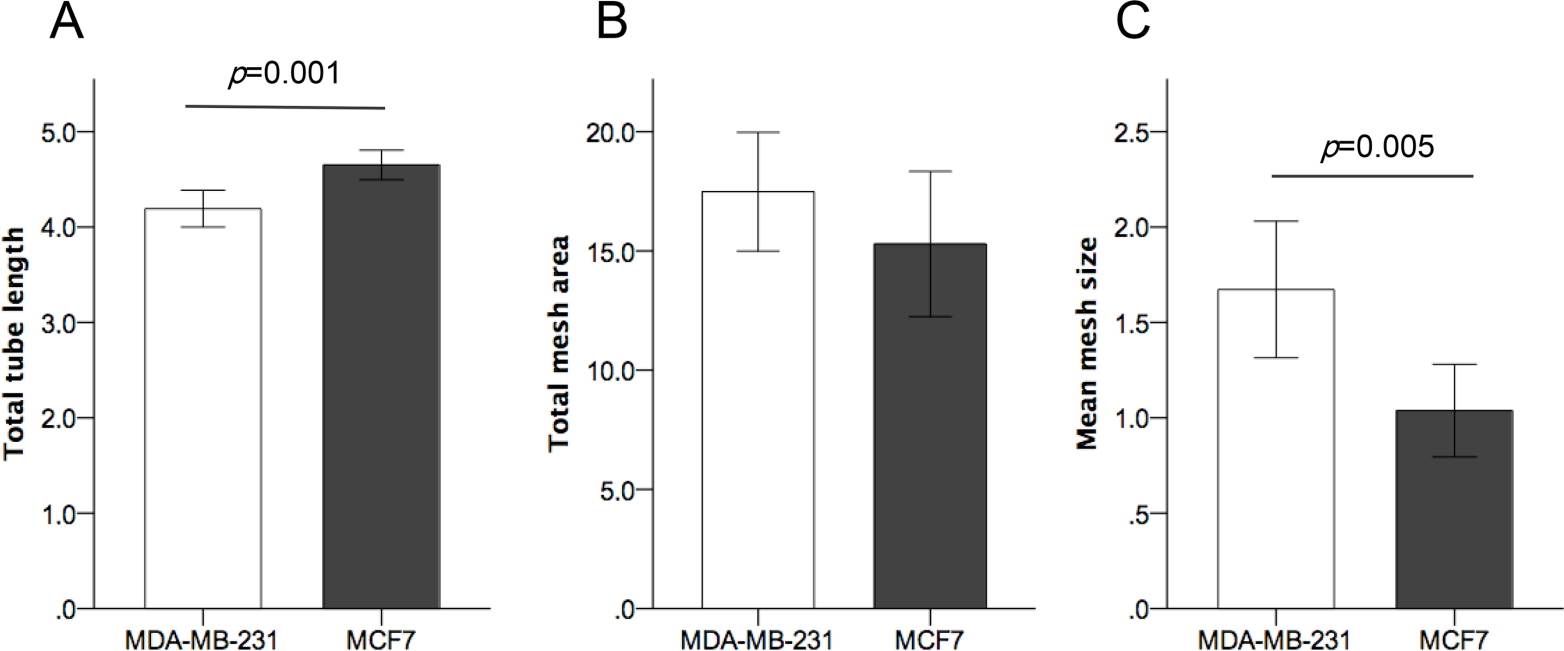
Comparison of angiogenic effects of conditioned media from MDA-MB-231 and MCF7 on endothelial cells. (A) Higher total tubule length induced by MCF-7 conditioned media (*p* = 0.001). (B) No significant differences in total mesh area. (C) Significantly larger mesh size (*p* = 0.005) induced by conditioned media from MDA-MB-231 breast carcinoma cells. All experiments were repeated three times (with eight biological replicates per condition). Units of length (tubule length and mesh size) and area (total mesh area) correspond to x10^4^ pixels.

To explore the *in vitro* angiogenic effects of miR-20a in breast cancer cells we first analyzed the baseline expression of miR-20a and angiogenic factors in MCF7 and MDA-MB-231 breast cancer cell lines, representing luminal and basal-like breast cancer subtypes. We found a significantly higher expression of miR-20a, VEGFA and THBS1 in MDA-MB-231 cells (S1 Table). Tube formation assay performed with conditioned media from MDA-MB-231 and MCF7 showed differences in capillary-like structure formation by EA.hy926 endothelial cells: total tube length was slightly smaller (*p* = 0.001) (Fig 1A) and mean mesh size was higher (*p* = 0.005) (Fig 1C) for MDA-MB-231 than for MCF7, while no significant differences were found for total mesh area (Fig 1B). These data, taken together with the higher levels of expression of angiogenic factors, are consistent with a different angiogenic potential of MDA-MB-231, a triple negative breast cancer cell line, able to form larger vascular meshes.

**Fig 2:**
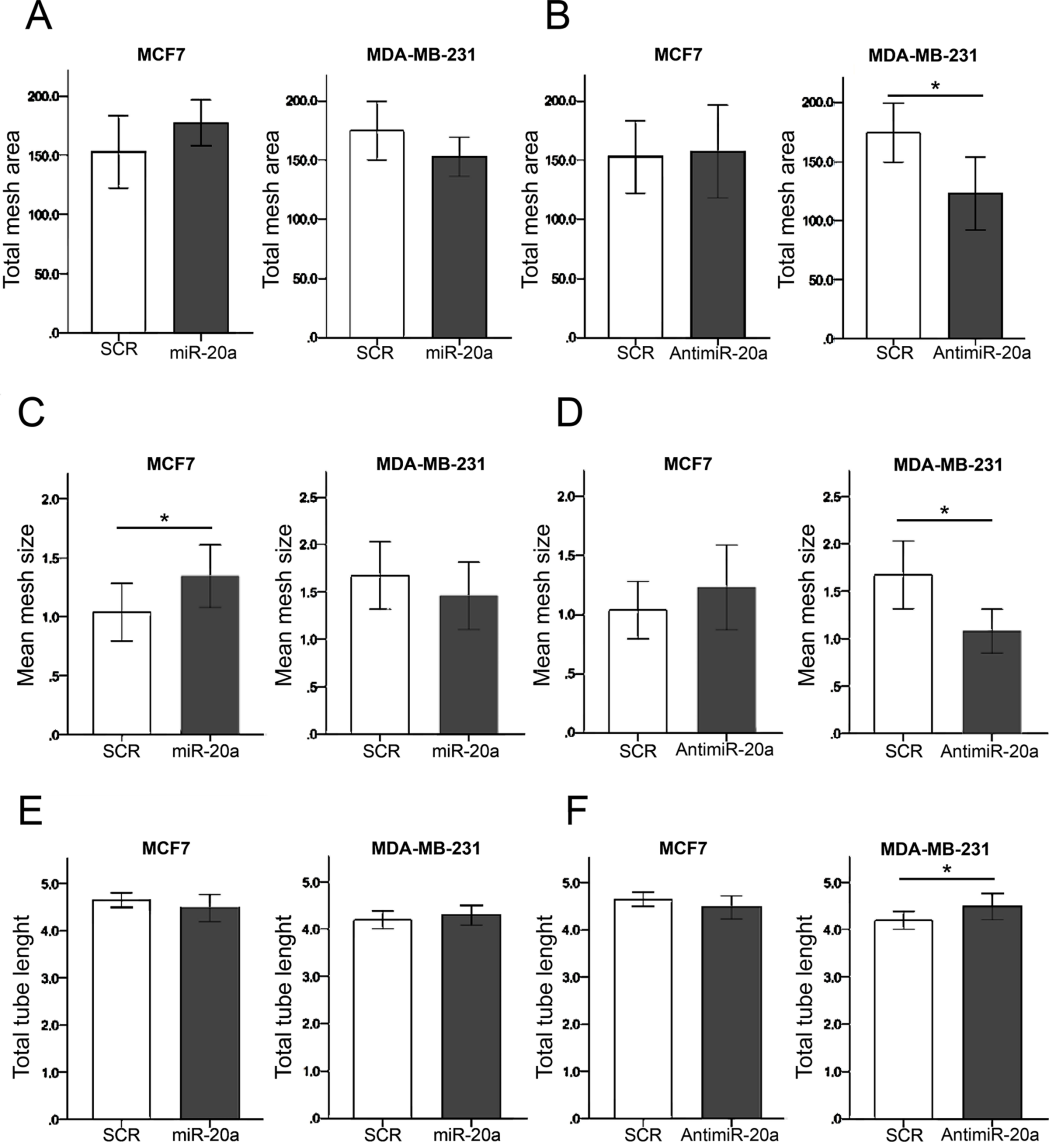
Effect of mir-20a changes on EA.hy926 capillary-like structure formation. (A) Total mesh area not modified by miR-20a overexpression in MCF7 and MDA-MB-231 breast carcinoma cells. (B) Transfection of MDA-MB-231 with anti-miR-20a induced a significant decrease in total mesh area (*p* = 0.001). (C) Mean mesh size of capillary-like structures formed by EA.hy926 was significantly increased by miR-20a transfection of MCF7 cells (*p* = 0.046). (D) A decrease of mean mesh size induced by anti-miR-20a transfection of MDA-MB-231 (*p* = 0.005). (E) No significant changes in total tubule length induced by miR-20a transfection. (F) Anti-miR20a transfection of MDA-MB-231 induced a slight, although significant (*p* = 0.049), increase of total tubule length. All experiments were repeated three times (with eight biological replicates per condition). Units of length (tubule length and mesh size) and area (total mesh area) correspond to x10^4^ pixels.

In order to determine the contribution of miR-20a to breast cancer angiogenesis, transfection of MDA-MB-231 and MCF7 with miR-20a mimic and anti-miR-20a was performed and conditioned media from transfected cell lines was used for tube formation assays with EA.hy926 endothelial cells. As shown in Fig 2 and Table 1, the effects of miR-20a were different in both cell lines: for MCF7 cells, with a low baseline expression of miR-20a, miR-20a mimic increased some angiogenic variables, while in MDA-MB-231, with a high baseline expression of miR-20a, angiogenic changes were induced by anti-miR-20a. Total mesh area was modified by miR-20a-mimic neither in MCF7 nor in MDA-MB-231 (Fig 2A), while antimiR-20a decreased total mesh area in MDA-MB-231 cells (*p* = 0.001) (Fig 2B). Modifications of mean mesh size were observed both after transfection of MCF7 with miR-20a-mimic, with a significant increase (*p* = 0.046)(Fig 2C), and after using anti-miR-20a in MDA-MB-231 cells, with a significant decrease of mesh size (*p* = 0.005) (Fig 2D). Total tube length was not modified by miR-20a-mimic (Fig 2E), and we only observed a slight increase with anti-miR-20a in MDA-MB-231 breast cancer cells (*p* = 0.049) (Fig 2F). Finally, the analysis of branching points (number of nodes) did not show any difference for MDA-MB-231 cells, while a borderline significant increase of them was observed after treatment with conditioned media from MCF7 cells transfected with anti-miR-20a (Table 1). The overexpression of miR-20a in MCF7 breast cancer cells did not change the mRNA expression of VEGFA (*p* = 0.20) or other angiogenic factors, such as PDGFA (*p* = 0.83) or CTGF (*p* = 0.83) (S2 Table). Also, no miR-20 related changes in protein expression of VEGFA were observed (S1 Fig). Transfection of miR-20a mimic did not significantly change the expression of other members of the cluster miR-17-92, except for a mild increase in miR-17 expression (S3 Table). In summary, miR-20a seems to modify the morphology of the vascular-like network, inducing a significant increase in the mean size of the meshes, but either without or with only slight changes in the total tube length and without changes in the expression of VEGFA or other angiogenic factors.

**Table 1:** Angiogenic *in vitro* effects of miR-20a in breast cancer cell lines (MCF7 and MDA-MB-231).

	Control^a^ (scramble)	miR-20a mimic^a^	*P*^b^	anti-miR-20a^a^	*P*^b^
**MDA-MB-231**					
**Total tube length**	41.92, 1.93	42.82, 2.02	0.34	44.82, 2.77	0.049
**Total mesh area**	1748.25, 249.03	1528.13, 165.36	0.07	1230.54, 309.64	0.001
**Mean mesh size**	16.72, 3.58	14.57, 3.55	0.29	10.80, 2.33	0.005
**Number of nodes**	1183, 141	1185, 106	0.64	1261, 182	0.50
**MCF7**					
**Total tube length**	46.51, 1.55	44.85, 2.88	0.14	44.81, 2.48	0.13
**Total mesh area**	1529.03, 304.59	1772.47, 191.80	0.11	1573.14, 391.51	0.79
**Mean mesh size**	10.38, 24.29	13.44, 2.65	0.046	12.31, 3.60	0.57
**Number of nodes**	1533, 150	1371, 189	0.10	1375, 154	0.05

^a^ Mean, SD. Units of length (tubule length and mesh size) and area (total mess area) correspond to x10^3^ pixels.

^b^ Comparison of each group with control (scramble); non-parametric Mann-Whitney test.

N = 8 for all observations.

**Fig 3:**
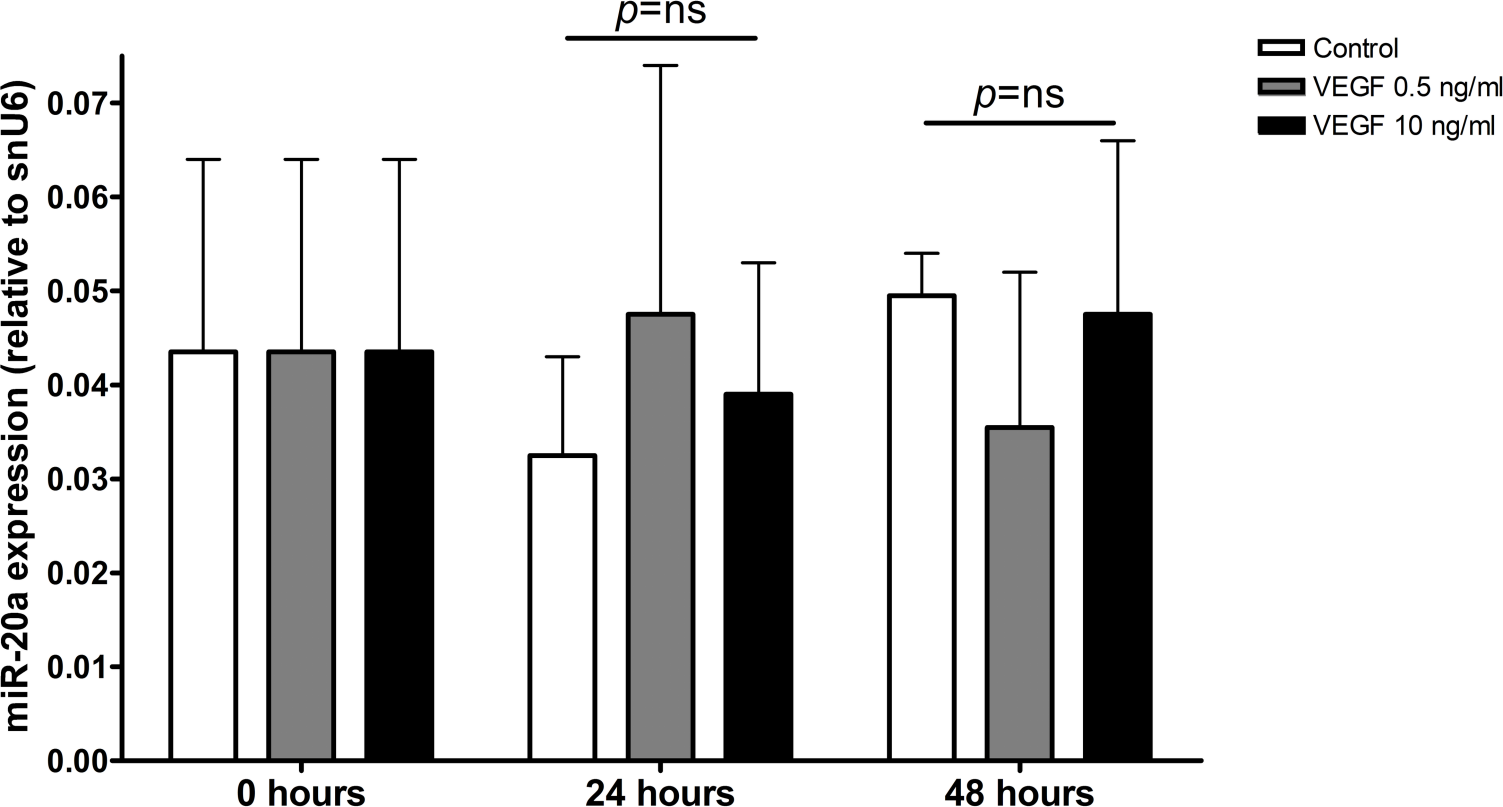
Effect of VEGFA exposure on miR-20a expression by MCF7 cells. No significant change of MCF7 miR-20a expression (relative to snU6 expression) after exposure to low (0.5 ng/mL) or high (10 ng/mL) recombinant human VEGFA concentrations (24 and 48 hours).

To further understand the relationship between VEGFA levels and miR-20a angiogenic effects, we performed additional experiments analyzing the potential modification of miR-20a expression by exposure to VEGFA. As shown in Fig 3, treatment of MCF7 cells with VEGFA at low and high concentrations (0.5 and 10 ng/mL) did not modify miR-20a expression, thereby suggesting that miR-20a expression is not related to VEFGA levels in the tumor environment. Additionally, to determine whether VEGFA was essential for the miR-20a angiogenic effect, angiogenesis assays were performed with conditioned media from MCF7 cells transfected with miR-20a and treated with or without aflibercept, a VEFG-trap with antiangiogenic effects [25]. As shown in S2 Fig no pro-angiogenic effect of miR-20a was observed in the presence of aflibercept, suggesting that miR-20a angiogenic effects are VEGFA-dependent. Finally, we evaluated endothelial cells proliferation in the same experimental model: a slight decrease of endothelial proliferation was observed when conditioned medium obtained from MCF7 cells transfected with miR-20a, while no significant changes in proliferation of endothelial cells were observed in the presence of aflibercept, thereby supporting the relevance of VEGFA exposure for miR-20a effects on endothelium (S3 Fig).

### Association of miR-20a expression with clinical characteristics and angiogenic marker expression in public databases

We analyzed the association between miR-20a expression and clinical characteristics of breast cancer using the TCGA breast cancer database (S4 Table). A significantly higher expression of miR-20a was found in estrogen receptor-negative tumors, and particularly in basal-like molecular subtype. The expression of miR-20a was also higher in luminal B tumors than in luminal A and HER2-enriched subtypes.

Although no previous data are available on the association of miR-17-92 expression and the vascular profile of breast carcinoma, the TCGA database was also used to explore the associations between miR-20a and the expression of angiogenic markers (S5 Table). A significant direct association was found for VEGFA and HIF1A, while a negative significant correlation was found for THBS1, CTGF and PDGFA.

### Expression of miR-20a in breast cancer and its association with clinical and pathological characteristics

In order to clinically confirm the relevance of miR-20a for breast cancer angiogenesis, its expression was examined in pre-treatment paraffin-embedded core biopsies from a series of breast cancer patients treated with neoadjuvant chemotherapy (S1 Dataset). The characterization of the angiogenic profile in patients of this group with enough available tissue (n = 86) has been previously described [9]. The expression of miR-20a was significantly higher in tumors with no expression of hormone receptors (HR), in triple negative breast cancer and in those cases with more extensive nodal involvement (cN2-3) (Table 2). Histological grade 3 was also associated with higher miR-20a expression. Expression levels, however, were not different among subgroups of patients defined by HER2 overexpression, or by clinical or pathological response to neoadjuvant chemotherapy (Table 2). No association was found between miR-20a expression and DFS (log-rank test; *p* = 0.62) or OS (log-rank test; *p* = 0.78). These findings were in agreement with those obtained from TCGA.

**Table 2:** Association of miR-20a expression and clinical-pathological characteristics of breast cancer patients (n = 95).

	N	miR-20a expression	*P*^a^
**Nodal stage**			
cN0-1	62	0.017 (0.003–1.970)	0.004
cN2-3	33	0.034 (0.006–0.165)	
**Grade**			
G1-2	36	0.016 (0.003–1.970)	0.041
G3	53	0.028 (0.005–0.165)	
**Hormone receptors**			
No	36	0.037 (0.005–1.970)	<0.001
Yes	59	0.016 (0.003–0.140)	
**HER2**			
No	68	0.022 (0.003–1.970)	0.46
Yes	27	0.027 (0.005–0.165)	
**Triple negative breast cancer**			
Yes	23	0.038 (0.007–1.970)	0.002
No	72	0.019 (0.003–0.165)	
**Clinical response**			
PR/CR	82	0.025 (0.003–1.970)	0.71
SD/PD	9	0.025 (0.003–0.165	
**pCR**			
No	20	0.030 (0.005–0.756)	0.17
Yes	74	0.021 (0.030–1.970)	

^a^ U Mann-Whitney

### Association of miR-20a with the angiogenic profile of breast cancer

**Fig 4:**
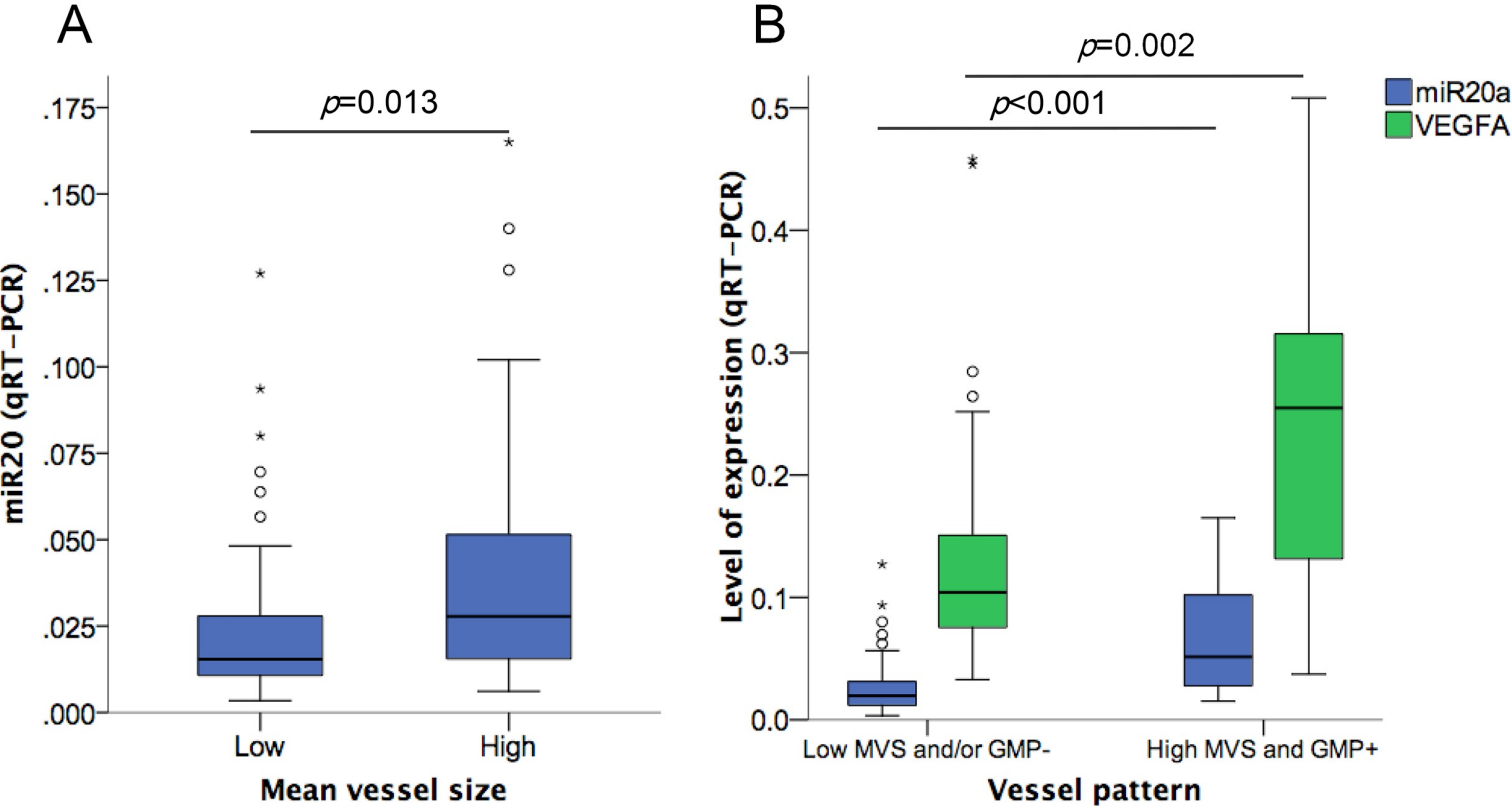
Association of miR-20a expression with vascular characteristics of breast cancer. (A) Association between high (over the median value) MVS and miR-20a expression in breast cancer (U Mann-Whitney; p = 0.013). (B) Association of high miR-20a (*p*<0.001) and VEGFA (*p* = 0.002) expression with a high-risk angiogenic profile (GMP+/MVS high).

As shown in Table 3, the expression of miR-20a was associated with the angiogenic profile of the tumor, showing a moderate but significant correlation with MVS (Rho: 0.276; *p* = 0.015). Breast carcinomas with high (over the median) mean vessel size showed a higher expression of miR-20a (U Mann-Whitney; *p* = 0.013) (Fig 4A). The analysis of the expression of other members of the cluster miR-17-92 in the same biopsies showed a high degree of correlation between them and with miR-20a (p<0.0001 in all cases) (S6 Table). However, no association of MVS with other members of miR-17-92 cluster was found (S7 Table). In order to further understand the basis for the association of miR-20a expression with MVS, we analyzed the correlations between miR-20a expression and the angiogenic biomarkers profile of breast cancer. We observed a significant correlation of its expression with VEGFA tumor levels (*p* = 0.004; correlation coefficient [CC]: 0.346). The expression of PDGFA was inversely correlated with miR-20a, while no differences were observed for THBS1, HIF1 and CTGF (Table 3). Thus, a higher miR-20a expression was associated with a distinct angiogenic profile characterized by a high vessel size pattern and high VEGFA expression.

**Table 3:** Correlation between miR-20a expression and the angiogenic characteristics of breast carcinomas.

	Correlation with miR-20a (Rho)	*P*^a^
**Vessel pattern (n = 77)**		
**MVD**	-0.164	0.153
**VA**	-0.022	0.853
**MVS**	0.276	0.015
**Angiogenic markers (n = 86)**		
**VEGFA**	0.346	0.004
**THBS1**	0.230	0.058
**PDGFA**	-0.276	0.022
**HIF1a**	-0.06	0.63
**CTGF**	-0.112	0.35

^a^ Spearman’s Rho

We previously described a VEGFA-related bad prognosis angiogenic profile characterized by high MVS and by the presence of GMP structures [9]. Breast tumors with this angiogenic profile (approximately, 28% of all cases) showed a particularly high expression of VEGFA [9] and miR-20a (*p*<0.0001) (Fig 4B) (S8 Table). This association did not seem to be derived only from the correlation between VEGFA and miR-20a expression, since miR-20a expression was an independent factor for its occurrence in a multivariate logistic regression model including VEGFA expression and breast cancer subtype (S9 Table).

## Discussion

We here show that the expression of miR-20a, a member of the miR-17-92 cluster, is associated with the angiogenic profile of breast cancer. *In vitro* data using conditioned media from breast cancer cell lines transfected with miR-20a suggest that miR-20a induces an increased vascular mesh size. The analysis of a clinical series consistently shows that a higher miR-20a expression is associated with a vascular pattern consisting in larger vessels and complex vascular structures (glomeruloid microvascular proliferations), together with high VEGFA expression.

Our results for the *in vitro* tube formation assay showing a significant increase in the mean size of the endothelial meshes, together with slight changes in the number of branching nodes and in endothelial proliferation, suggest that miR-20a modifies the characteristics of the vascular-like network and might contribute to the development of an abnormal angiogenic pattern in breast carcinomas. Although miR-20a expression was also associated with a distinct angiogenic pattern and an increased vascular size in pre-treatment biopsies, our *in vitro* findings are not exactly parallel to histological data since no change was observed in vascular density. Previous data on miR-17-92 cluster regulation suggest that angiogenic modulation occur in the context of a high VEGF environment. The transcriptional control of miR-17-92 in endothelial cells seems to be VEGF-dependent [22], and in a mouse model with endothelial miR-17-92 deletion, the participation of the cluster miR-17-92 in VEGF-dependent tumor angiogenesis was demonstrated. Although our model is focused in the effects of miR-20a expression on tumor cells, and not on endothelial cells, we found that miR-20a modifies neither vascular density nor expression of angiogenic factors, similarly to the results obtained after transfection of the whole cluster miR-17-92 [21,22]. However, the effects of miR-17-92 on breast carcinoma biology are pleiotropic [26] and might also include indirect effects on angiogenesis or metastatic potential [27], potentially related to extracellular matrix changes [28], complex modulation of ER pathways [29] or regulation of tumor-suppressor genes [30,31]. Additionally, previous reports have shown different effects of miR-17/20 on endothelial cells when analyzed either *in vitro* or *in vivo* and a predominantly antiangiogenic effect in colorectal cancer [32,33], which suggest that angiogenesis regulation by miR-17-92 is both microenvironment- and cell type-dependent. Interestingly, our data show that VEGF levels do not modify the expression of miR-20a and, at the same time, that treatment with a VEGFA trap inhibits the angiogenesis induced by luminal breast cancer cells transfected with miR-20a. Additionally, the mild decrease of endothelial proliferation induced by overexpression of miR-20a in tumor cells was not observed in the presence of aflibercept. Taken together, these findings are consistent with the notion that miR-20a-related modulation of breast cancer angiogenesis is dependent on VEGFA.

Our clinical data further support the experimental results. We found higher levels of miR-20a in ER negative tumors, and especially in triple negative breast cancer, in agreement with previous analysis of miRNA expression profiles in this subtype [34], for which miR-17-92 expression has been proposed as a distinctive characteristic [35]. The association of miR-20a expression with grade 3 and especially with higher nodal involvement has not been shown previously [36], but is also consistent with our analysis of TCGA public database. The higher expression of miR-20a in MDA-MB-231 (triple negative) when compared with MCF7 (luminal) cell line also reflects the clinical results and has been previously reported [37].

As far as we know, no previous work has analyzed the correlation of miR-17-92 expression with the vascular pattern in breast carcinomas. Our finding of the association between miR-20a expression and a high risk vascular profile defined by larger size vessels and by the presence of GMP is thus a novel finding, which warrants further study of miR-20a angiogenic effects on breast cancer. Clinical and experimental data suggest that these effects occur in the context of a high VEGFA-tumor microenvironment [38]. The induction by miR-20a of an abnormal vascular maturation characterized by higher size vessels might be also prognostically relevant [9,14].

These results do not seem to be merely explained by the association with the triple negative subtype, since miR-20a expression was still an independent factor for the occurrence of this vascular profile in the multivariate analysis, and only one in four of our patients had a triple negative breast cancer. Our experimental results in MCF7 cells might suggest the involvement of miR-20a in luminal breast cancer angiogenesis, although the correlation of clinical and in vitro data is incomplete and the level of miR-20a is usually low in this tumor subtype.

Our work has several limitations, the main one being the small sample size. Although experimental results are globally consistent with clinical and pathological data, the effects were moderate and further mechanistic insights might also be obtained with a more comprehensive analysis of angiogenic markers and endothelial changes (apoptosis, proliferation, migration) or with *in vivo* experiments focusing on the angiogenic effects of miR-20a. Also, part of our findings, such as the increased microvessel size, might be caused by changes in pericyte coverage [39], a possibility that was not addressed in this study. Additionally, our in vitro experimental model allows neither the understanding of the physiological consequences of a high vascular size nor an evaluation of other angiogenic mechanisms mediated by tumor microenvironment.

However, these findings, if confirmed, raise the possibility for the development of angiogenesis-related miRNAs as surrogate biomarkers of a VEGF-dependent high-risk angiogenic profile. In particular, circulating miRNA or miRNA determined in other body fluids are easily determined and might be used to prognostically stratify breast cancer patients [40] or as screening tools for selection of patients for trials with anti-angiogenic drugs [41]. In fact, other members of the cluster miR-17-92 have been included in a breast cancer diagnostic signature of circulating miRNA [42], although no data are available regarding its association with tumor angiogenesis. Additionally, the data showing an almost complete abrogation of vessel formation by aflibercept in the experimental model of MCF7 cells transfected with miR-20a might suggest the potential value of high miR-20a expression as a marker of sensitivity to antiangiogenic strategies, which warrants further studies to evaluate its predictive value.

## Conclusions

Expression of miR-20a, a member of the miR-17-92 cluster, in breast cancer cells induces the development of an abnormal vascular mesh. In agreement with experimental data, its expression in invasive breast carcinomas is associated with a VEGF-related high-risk angiogenic profile characterized by larger vessels and glomeruloid microvascular proliferations. Further clinical and experimental validation of these findings might be relevant for the use of miR-20a as a biomarker for selection of antiangiogenic treatments for breast cancer and as a potential new angiogenic target.

## Supporting information

S1 Table MiR-20a expression in breast carcinoma cell lines. Differences in level of expression (median, interquartile range) of miR-20a and angiogenic factors between MCF7 and MDA-MB-231 breast carcinoma cell lines.
(DOCX)

S2 Table MiR-20a effect on angiogenic factors expression. Comparison of the expression (mRNA) of VEGFA and other angiogenic factors (median, interquartile range) after transfection with miR-20a mimics vs. control in MDA-MB-231 and MCF7.
(DOCX)

S3 Table MiR-17-92 expression changes induced by miR-20a transfection. Comparison of the expression levels (median) of different members of cluster miR-17-92 after transfection with miR-20a mimics vs. control in MDA-MB-231 and MCF7.
(DOCX)

S4 Table Clinical associations of miR-20a in TCGA. External validation (TCGA public database) of associations between miR-20a expression level and breast cancer clinical characteristics.
(DOCX)

S5 Table Association of angiogenic factor expression with miR-20a in TCGA. External validation (TCGA public database) of associations between miR-20a and the expression of angiogenic biomarkers in breast cancer.
(DOCX)

S6 Table MiR-17-92 expression in breast cancer. Correlation of levels of expression between members of miR-17-92 cluster in breast cancer biopsies (Spearman’s Rho; p values).
(DOCX)

S7 Table MiR-17-92 and vessel size. Association of miR-17-92 cluster with breast cancer mean vessel size (Spearman’s Rho).
(DOCX)

S8 Table MiR-20a and vascular pattern. Association of miR-20a expression (median, interquartile range) with high-risk angiogenic profile (MVS high/GMP+).
(DOCX)

S9 Table Multivariate model. Multivariate model for high-risk angiogenic profile (high vessel size and GMP) in untreated breast cancer.
(DOCX)

S1 Fig Analysis of VEGFA protein expression in MCF7 and MDA-MB-231 cells transfected with miR-20a vs. antimiR-20a vs. control. (A) Western-blot bands of VEGF and β-actin used for quantification. (B) Box-plot of VEGFA expression (normalized to β-actin) in both cell lines. No differences were found between conditions (Kruskal-Wallis Test; *p* = 0.148 and *p* = 0.177 respectively).
(TIF)

S2 Fig VEGFA-dependence of angiogenic effects of conditioned media from MCF7 transfected with miR-20a. Photomicrographs showing the abrogation of tubule formation (EA.hy926 endothelial cells) induced by conditioned media from MCF7 cells transfected with miR-20a after exposure to aflibercept (500 μg/ml).
(TIF)

S3 Fig Effects of miR-20a changes in MCF7 on EA.hy926 proliferation. Significant increase in proliferation index (XTT assay) of EA.hy926 endothelial cells cultured on conditioned media from MCF7 breast cancer cells transfected with miR-20a (vs. scrambled control). Proliferation increase by miR-20a was not observed in the presence of aflibercept (VEGFA trap). *** *p*<0.001; ns: non significant.
(TIF)

S1 Dataset Clinical, pathological and angiogenesis data of breast cancer patients with available data of angiogenic factors expression and/or vessel profile evaluation (n = 95). (XLSX)
