# Supplementary material for: Angiogenic role of miR-20a in breast cancer
Source: PLoS One. 2018 Apr 4;13(4):e0194638. doi: 10.1371/journal.pone.0194638 (PMC5884522; doi:10.1371/journal.pone.0194638)
Supplement: S1 Table — Differences in level of expression (median, interquartile range) of miR-20a and angiogenic factors between MCF7 and MDA-MB-231 breast carcinoma cell lines. (DOCX) [file pone.0194638.s001.docx]

**S1 Table. MiR-20a expression in breast carcinoma cell lines.** Differences in level of expression (*median, interquartile range*) of miR-20a (relative to snU6) and angiogenic factors (mRNA expression relative to ACTB) between MCF7 and MDA-MB-231 breast carcinoma cell lines.

| N=9 | **MCF7** | **MDA-MB-231** | ***P*^a^** |
| --- | --- | --- | --- |
| **miR-20a** | 0.071 (0.070-0.082) | 0.15 (0.15-0.21) | <0.001 |
| ***VEGFA*** | 0.0017 (0.0016-0.0021) | 0.0128 (0.0127-0.0154) | <0.001 |
| ***THBS1*** | 0.028 (0.021-0.037) | 0.213 (0.192-0.239) | <0.001 |
| ***PDGFA*** | 0.0012 (0.0009-0.0013) | 0.0005 (0.0004-0.0005) | <0.001 |
| ***HIF1A*** | 0.0107 (0.0102-0.0168) | 0.016 (0.015-0.016) | 0.26 |

^a^ Mann-Whitney U test
