# Supplementary material for: Angiogenic role of miR-20a in breast cancer
Source: PLoS One. 2018 Apr 4;13(4):e0194638. doi: 10.1371/journal.pone.0194638 (PMC5884522; doi:10.1371/journal.pone.0194638)
Supplement: S2 Table — Comparison of the expression (mRNA) of VEGFA and other angiogenic factors (median, interquartile range) after transfection with miR-20a mimics vs. control in MDA-MB-231 and MCF7. (DOCX) [file pone.0194638.s002.docx]

**S2 Table. MiR-20a effect on angiogenic factors expression.** Comparison of the expression (mRNA) of VEGFA and other angiogenic factors (*median, interquartile range*) after transfection with miR-20a mimics vs. control in MDA-MB-231 and MCF7.

| N=9 | **Control (scramble)** | **miR-20a mimic** | ***P*^a^** |
| --- | --- | --- | --- |
| **MDA-MB-231** | | | |
| VEGFA | 0.0372 (0.03620-0.03790) | 0.0327 (0.03210-0.03580) | 0.10 |
| PDGFA | 0.00179 (0.0003-0.00208) | 0.000738 (0.000686-0.00205) | 1.00 |
| CGTF | 0.0372 (0.03440-0.03960) | 0.0426 (0.03960-0.04500) | 0.10 |
| **MCF7** | | | |
| VEGFA | 0.00389 (0.00383-0.00435) | 0.00437 (0.004080-0.004510) | 0.20 |
| PDGFA | 0.00229 (0.00160-0.00279) | 0.00165 (0.00122-0.003010) | 1.00 |
| CGTF | 0.00012 (0.00011-0.00015) | 0.00013 (0.000092-0.000184) | 1.00 |

^a^ Mann-Whitney U test
