# Supplementary material for: Angiogenic role of miR-20a in breast cancer
Source: PLoS One. 2018 Apr 4;13(4):e0194638. doi: 10.1371/journal.pone.0194638 (PMC5884522; doi:10.1371/journal.pone.0194638)
Supplement: S3 Table — Comparison of the expression levels (median) of different members of cluster miR-17-92 after transfection with miR-20a mimics vs. control in MDA-MB-231 and MCF7. (DOCX) [file pone.0194638.s003.docx]

**S3 Table. MiR-17-92 expression changes induced by miR-20a transfection.** Comparison of the expression levels (median) of different members of cluster miR-17-92 after transfection with miR-20a mimics vs. control in MDA-MB-231 and MCF7.

|  | **MDA-MB-231** | | | **MCF7** | | |
| --- | --- | --- | --- | --- | --- | --- |
| **microRNA** | **Scrambled** | **miR-20a** | ***P*** | **Scrambled** | **miR-20a** | ***P*** |
| miR-17 | 0.1022  0.0725  0.0974 | 0.0944  0.1044  0.1150 | 0.275 | 0.0307  0.0317  0.0373 | 0.0497  0.0597  0.0377 | 0.049 |
| miR-18a | 0.0144  0.0096  0.0108 | 0.0100  0.0088  0.0125 | 0.513 | 0.0039  0.0035  0.0048 | 0.0042  0.0051  0.0027 | 0.827 |
| miR-19b | 0.1037  0.0754  0.0884 | 0.0778  0.0623  0.1077 | 0.827 | 0.0443  0.0402  0.0524 | 0.0459  0.0517  0.0284 | 0.827 |
| miR-20a | 0.0788  0.0567  0.0743 | 7.3871  7.2854  9.8151 | 0.049 | 0.0260  0.0265  0.0317 | 5.0806  6.0629  4.0982 | 0.049 |
| miR-92a | 0.0863  0.0668  0.0751 | 0.0756  0.0723  0.0857 | 0.827 | 0.0263  0.0290  0.0341 | 0.0313  0.0443  0.0251 | 0.827 |
