# Supplementary material for: Angiogenic role of miR-20a in breast cancer
Source: PLoS One. 2018 Apr 4;13(4):e0194638. doi: 10.1371/journal.pone.0194638 (PMC5884522; doi:10.1371/journal.pone.0194638)
Supplement: S4 Table — External validation (TCGA public database) of associations between miR-20a expression level and breast cancer clinical characteristics. (DOCX) [file pone.0194638.s004.docx]

**S4 Table. Clinical associations of miR-20a in TCGA.** External validation (TCGA public database) of associations between miR-20a level of expression and breast cancer clinical characteristics.

| N=780 | **n** | **miR-20a**  Median, IQR | ***P*** |
| --- | --- | --- | --- |
| **Estrogen receptor**  Positive  Negative | 601  179 | 122.1, 81.1-193.2  269.1, 168.4-511.4 | <0.0001 |
| **HER2 amplification**  Yes  No | 114  652 | 136.5, 89.9-224.8  145.0, 88.2-145.2 | 0.217 |
| **Tumor subtype (PAM50)**  Basal-like  HER2-enriched  Luminal A  Luminal B | 98  58  231  127 | 376.5, 220.5-778.7  126.8, 86.9-212.3  109.2, 71.3-174.0  168.0, 110.7-245.9 | <0.0001 |
| **pN**  Negative  Positive | 385  406 | 145.9, 94.2-262.9  140.5, 85.6-227.9 | 0.077 |
