# Supplementary material for: Angiogenic role of miR-20a in breast cancer
Source: PLoS One. 2018 Apr 4;13(4):e0194638. doi: 10.1371/journal.pone.0194638 (PMC5884522; doi:10.1371/journal.pone.0194638)
Supplement: S5 Table — External validation (TCGA public database) of associations between miR-20a and the expression of angiogenic biomarkers in breast cancer. (DOCX) [file pone.0194638.s005.docx]

**S5 Table. Association of angiogenic factor expression with miR-20a in TCGA.** External validation (TCGA public database) of associations between miR-20a and the expression of angiogenic biomarkers in breast cancer.

| N=685 | **Correlation with miR-20a (Rho)** | ***P*** |
| --- | --- | --- |
| **VEGFA** | 0.202 | <0.0001 |
| **THBS1** | -0.247 | <0.0001 |
| **PDGFA** | -0.093 | 0.015 |
| **HIF1a** | 0.113 | 0.003 |
| **CTGF** | -0.178 | <0.0001 |
