# Supplementary material for: Angiogenic role of miR-20a in breast cancer
Source: PLoS One. 2018 Apr 4;13(4):e0194638. doi: 10.1371/journal.pone.0194638 (PMC5884522; doi:10.1371/journal.pone.0194638)
Supplement: S6 Table — Correlation of levels of expression between members of miR-17-92 cluster in breast cancer biopsies (Spearman’s Rho; p values). (DOCX) [file pone.0194638.s006.docx]

**S6 Table.** **MiR-17-92 expression in breast cancer.** Correlation of levels of expression between members of miR-17-92 cluster in breast cancer biopsies (Spearman’s Rho; p values).

|  | **miR-20a** | **miR-19a** | **miR-18a** | **miR-17** | **miR-92a** | **miR-106b** |
| --- | --- | --- | --- | --- | --- | --- |
| **miR-20a** | ---- | .927  <0.0001 | .577  <0.0001 | .679  <0.0001 | .472  <0.0001 | .636  <0.0001 |
|  |  |  |  |  |  |  |
| **miR-19a** | .927  .000 | ---- | .604  .000 | .676  .000 | .447  .000 | .688  .000 |
|  |  |  |  |  |  |  |
| **miR-18a** | .577  .000 | .604  .000 | ---- | .631  .000 | .499  .000 | .565  .000 |
|  |  |  |  |  |  |  |
| **miR-17** | .679  .000 | .676  .000 | .631  .000 | ---- | .679  .000 | .620  .000 |
|  |  |  |  |  |  |  |
| **miR-92a** | .472  .000 | .447  .000 | .499  .000 | .679  .000 | ---- | .595  .000 |
|  |  |  |  |  |  |  |
| **miR-106b** | .636  .000 | .688  .000 | .565  .000 | .620  .000 | .595  .000 | ---- |
|  |  |  |  |  |  |  |
