# Supplementary material for: Angiogenic role of miR-20a in breast cancer
Source: PLoS One. 2018 Apr 4;13(4):e0194638. doi: 10.1371/journal.pone.0194638 (PMC5884522; doi:10.1371/journal.pone.0194638)
Supplement: S7 Table — Association of miR-17-92 cluster with breast cancer mean vessel size (Spearman’s Rho). (DOCX) [file pone.0194638.s007.docx]

**S7 Table**. **MiR-17-92 and vessel size.** Association of miR-17-92 cluster with breast cancer mean vessel size (Spearman’s Rho).

| **N=77** | **MVS** |
| --- | --- |
| **miR-20a** | **.276** |
|  | **P=0.015** |
| **miR-19a** | .209 |
|  | P=0.068 |
| **miR-18a** | .075 |
|  | P=0.516 |
| **miR-17** | .128 |
|  | P=0.282 |
| **miR-92a** | -.051 |
|  | P=0.668 |
| **miR-106** | .188 |
|  | P=0.120 |

MVS: median vessel size.
