# Supplementary material for: Angiogenic role of miR-20a in breast cancer
Source: PLoS One. 2018 Apr 4;13(4):e0194638. doi: 10.1371/journal.pone.0194638 (PMC5884522; doi:10.1371/journal.pone.0194638)
Supplement: S8 Table — Association of miR-20a expression (median, interquartile range) with high-risk angiogenic profile (MVS high/GMP+). (DOCX) [file pone.0194638.s008.docx]

**S8 Table. MiR-20a and vascular pattern.** Association of miR-20a expression (median, interquartile range) with high-risk angiogenic profile (MVS high/GMP+)

| **N=69** | **Non-high risk profile (GMP- and/or MVS low)**  **N=51** | **High risk profile (GMP+ and MVS high)**  **N=18** | ***P*** |
| --- | --- | --- | --- |
| **miR-20** | 0.0177 (0.0118-0.0313) | 0.0514 (0.0271-0.1085) | <0.001 |
