# Supplementary material for: Angiogenic role of miR-20a in breast cancer
Source: PLoS One. 2018 Apr 4;13(4):e0194638. doi: 10.1371/journal.pone.0194638 (PMC5884522; doi:10.1371/journal.pone.0194638)
Supplement: S9 Table — Multivariate model for high-risk angiogenic profile (high vessel size and GMP) in untreated breast cancer. (DOCX) [file pone.0194638.s009.docx]

**S9 Table. Multivariate model.** Multivariate model for high-risk angiogenic profile (high vessel size and GMP) in untreated breast cancer

| **Covariates** | **OR** | **95% CI** | ***P*** |
| --- | --- | --- | --- |
| miR-20a | 6.153 | 1.436-26.375 | **0.014** |
| VEGFA | 3.913 | 0.989-15.475 | 0.052 |
| Triple negative breast cancer | 2.959 | 0.723-12.117 | 0.132 |
