# Supplementary figures and images for: Angiogenic role of miR-20a in breast cancer
Source: PLoS One. 2018 Apr 4;13(4):e0194638. doi: 10.1371/journal.pone.0194638 (PMC5884522; doi:10.1371/journal.pone.0194638)

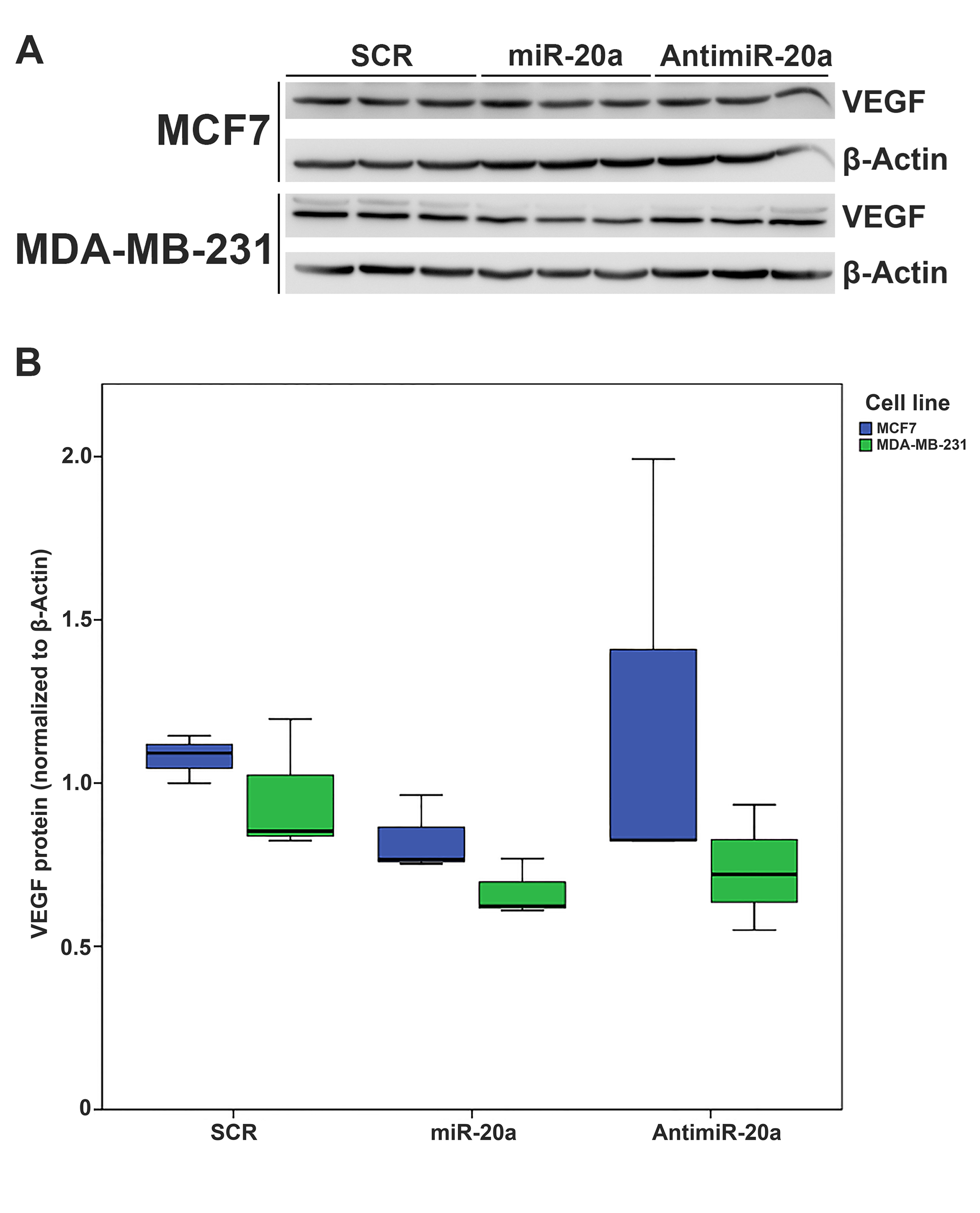

Supplement: S1 Fig — (A) Western-blot bands of VEGF and β-actin used for quantification. (B) Box-plot of VEGFA expression (normalized to β-actin) in both cell lines. No differences were found between conditions (Kruskal-Wallis Test; p = 0.148 and p = 0.177 respectively). (TIF) [file pone.0194638.s010.tif]

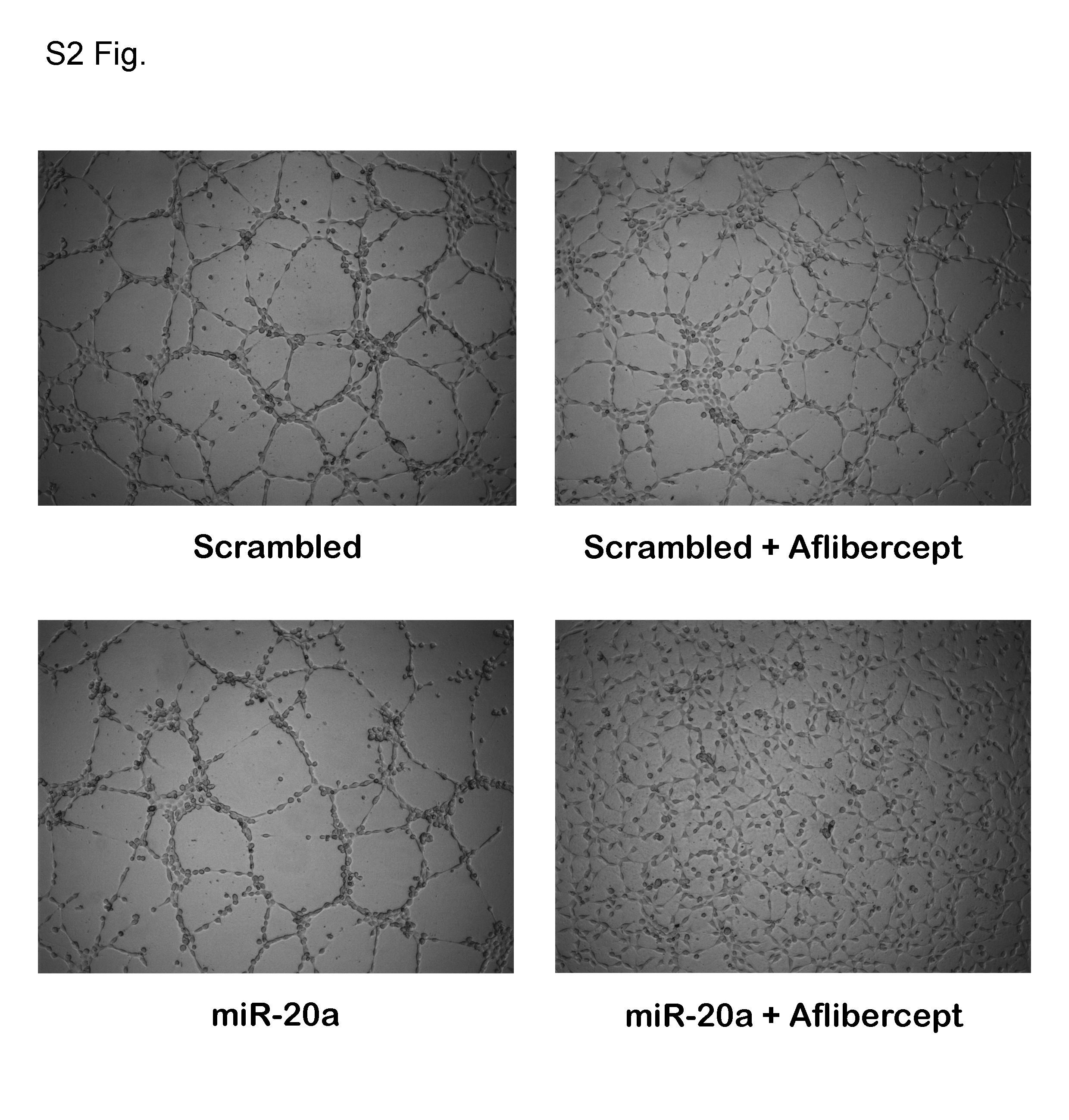

Supplement: S2 Fig — Photomicrographs showing the abrogation of tubule formation (EA.hy926 endothelial cells) induced by conditioned media from MCF7 cells transfected with miR-20a after exposure to aflibercept (500 μg/ml). (TIF) [file pone.0194638.s011.tif]

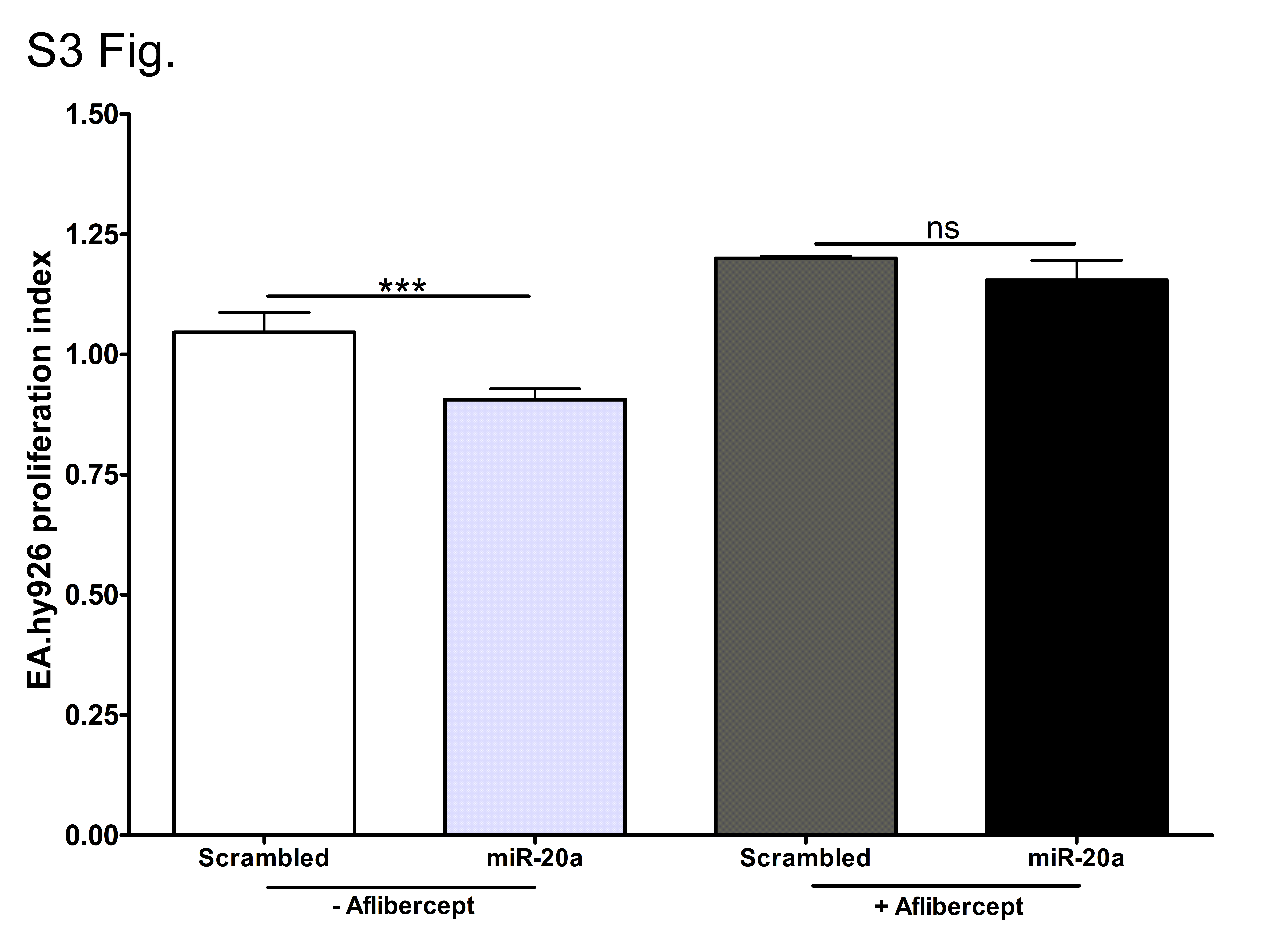

Supplement: S3 Fig — Significant increase in proliferation index (XTT assay) of EA.hy926 endothelial cells cultured on conditioned media from MCF7 breast cancer cells transfected with miR-20a (vs. scrambled control). Proliferation increase by miR-20a was not observed in the presence of aflibercept (VEGFA trap). *** p<0.001; ns: non significant. (TIF) [file pone.0194638.s012.tif]
